# Distribution of the soft tick *Carios vespertilionis* in lowlands and low mountain regions of Germany

**DOI:** 10.1007/s10493-023-00822-2

**Published:** 2023-07-27

**Authors:** Anja Fritzsche, Stefan Zaenker, Jannis Gottwald, Renate Keil, Christian Zaenker, Michael Bröker, Lidia Chitimia-Dobler

**Affiliations:** 1 Institute of Animal Ecology and Nature Education, Laubach/Gonterskirchen, Germany; 2 Hesse Federation for Cave and Karst Research, Fulda, Germany; 3 tRackIT Systems GbR, Marburg, Germany; 4 Sandstraße 7, 30629 Hannover 5 Pappelweg 30, 35041 Marburg, Germany; 5 Pappelweg 30, 35041 Marburg, Germany; 6 Bundeswehr Institute of Microbiology, Neuherbergstrasse 11, 80937 Munich, Germany

**Keywords:** Bats, *Carios* (*Argas) vespertilionis*, Germany, Tick map

## Abstract

In Germany, the knowledge about ticks infesting bats is limited, and is restricted only to a few studies, most of them dating back decades. To further improve our knowledge on ticks parasitising bats, healthy and sick bats in central Germany were examined for ticks. In total 519 larvae and one nymph of *Carios vespertilionis* were collected from nine bat species: *Eptesicus nilssonii*, *Eptesicus serotinus*, *Myotis daubentonii*, *Myotis myotis*, *Nyctalus leisleri*, *Pipistrellus nathusii*, *Pipistrellus pygmaeus*, *Pipistrellus pipistrellus*, and *Vespertilio murinus*. Either the presence of *C. vespertilionis* was new for some areas or it was confirmed in some federal states in central Germany. The infestation rate was mostly low (n = 1–5 larvae/bat). However, in two cases a high number of ticks was observed. The highest infestation of 97 *C*. *vespertilionis* larvae was recorded on one Parti-coloured bat (*V. murinus*).

## Introduction

Ticks are obligate blood-feeding arthropods, with 950 living species grouped into three families: Ixodidae (726 species), Argasidae (223 species) and Nuttalliellidae (a single species) (Guglielmone et al. 2023; Mans et al. 2021). Argasidae (soft ticks) are more diverse in subtropical and tropical areas with more than 70 species being bat-specialists (Guglielmone et al. 2010). The family Argasidae is divided into two subfamilies: Argasinae and Ornithodorinae. Two soft tick species are known to regularly infest European bats: *Carios vespertilionis* is the most widespread species, whereas *Secretargas transgariepinus* is known from only a few locations in the central and western part of the Mediterranean Basin (Sandor et al. 2019). *Carios vespertilionis*, formerly known as *Argas vespertilionis* (Mans et al. 2021), is widely distributed between the Palearctic Region and South Africa. In total 42 host species were reported (Sándor et al. 2021). The main hosts are: common pipistrelle (*Pipistrellus pipistrellus*), Nathusius’s pipistrelle (*P*. *nathusii*), Kuhl’s pipistrelle (*P*. *kuhlii*), common noctule (*Nyctalus noctula*), whiskered bat (*Myotis mystacinus*), serotine bat (*Eptesicus serotinus*), and parti-coloured bat (*Vespertilio murinus*) (Sándor et al. 2021).

*Carios vespertilionis* (short-legged bat tick) and *Argas reflexus* (pigeon tick) are the only soft tick species in Germany and have been reported in many German Federal States. *Carios vespertilionis* has been reported for the first time in 1906 in Bremen-Vegesack, northern Germany, and is the only bat-associated soft tick (Voigt and Oudemanns 1906). There are only a few reports about its distribution in Walter (1992) summarised *C*. *vespertilionis* hosts (totally 12 bat species) in nine Federal States. In Bavaria (southern Germany), 11 bat species were reported as hosts for *C*. *vespertilionis*, with *P*. *pipistrellus* being the primary host (Rupp et al. 2004).

*Carios vespertilionis* parasitises bats in different types of transient colonies (e.g., maternal colonies) in all possible roost types, such as attics, burrows, hollow trees, and caves (Siuda et al. 2009). *Carios vespertilionis* can also attack humans and is considered ‘highly aggressive’ (Hoogstraal 1985; Estrada-Peña and Jongejan 1999). Jaenson et al. (1994) observed severe skin reactions with fever, ulceration, erythema, and edema on the legs and arms of two persons who had been bitten by *C. vespertilionis* nymphs, near Stockholm. This tick was reported as a vector for several pathogens such as viruses (e.g., tick-borne encephalitis virus, Issyk-Kul virus), bacteria (e.g., *Coxiella burnetii*, *Ehrlichia*, *Rickettsia*, and *Borrelia* species), and piroplasmids (*Babesia* spp.) (Sándor et al. 2021; Tompa et al. 2023).

In this study, we describe the distribution of *C*. *vespertilionis* in central Germany collected from bats caught with mist nets and sick or debilitated bats found on the ground.

## Materials and methods

**Fig. 1:**
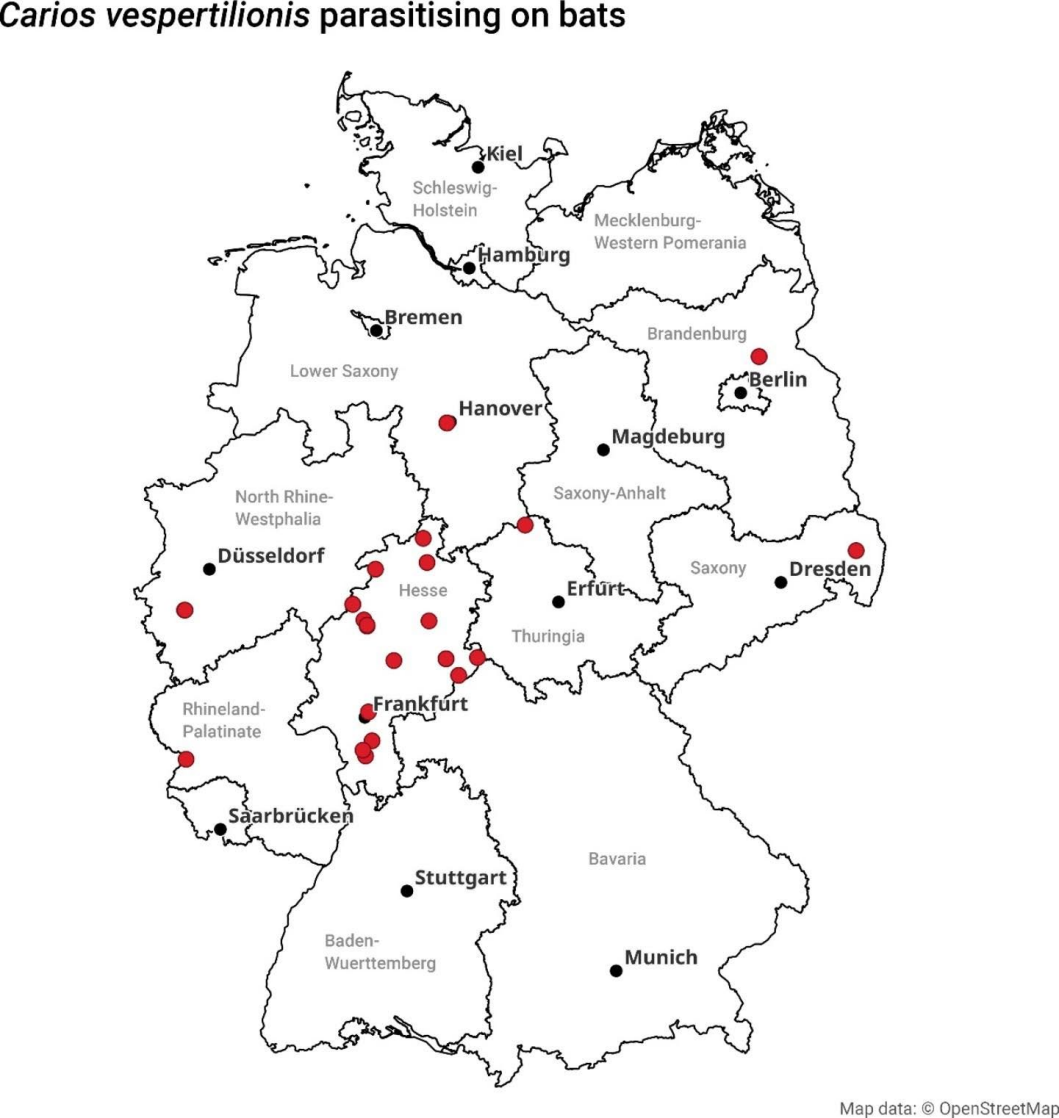
Recorded locations of *Carios vespertilionis* in central Germany: red dots, location of ticks; black dots, cities

Ticks were collected from captured bats or bats delivered to animal rescue stations across six German federal states: Lower Saxony, Brandenburg, Saxony, North Rhine-Westphalia, Hesse, and Rhineland-Palatinate (Fig. 1). Ethical approval to catch bats had been issued by local Nature Conservation Authorities.

Bat ticks were collected from June to September 2021 and in August 2022, in six federal states in central Germany. Ticks were collected from bats caught with mist nets, and sick or debilitated bats coddled up in two volunteer bat rescue stations in Fulda city, eastern Hesse from 2002 to 2009 and in the urban region of Hanover, Lower Saxony, during summer 2022 and early 2023.

The bats were searched for ticks by brushing their fur with a small soft brush (to reduce the risk of possible infections of the handling person). The ticks were removed using fine-tipped tweezers and preserved in microtubes containing 70% ethanol, labelled according to each host. The ticks were identified using morphological characters according to Feider (1965).

## Results

In total 519 *C*. *vespertilionis* larvae and one nymph were collected from 47 bats (Tables 1 and 2). Those bats belonged to nine species: *Eptesicus nilssonii*, *E. serotinus*, *Myotis daubentonii*, *M. myotis*, *Nyctalus leisleri*, *Pipistrellus nathusii*, *P. pygmaeus*, *P. pipistrellus*, and *Vespertilio murinus*. In the current study, *P. pipistrellus* was the dominant bat species from which *C. vespertilionis* larvae were collected. Generally, a few *C. vespertilionis* larvae were collected from bats, except for two cases: a *P. nathusii* was infested with 72 larvae and a *V*. *murinus* was infested with 97 larvae (Tables 1 and 2).

**Table 1:** *Carios vespertilionis* collected from healthy bats caught by mist nets in central Germany

Date	Location	Federal state	Bat species	No. larvae
June 2021	Lahntal	Hesse	*Nyctalus leisleri*	3
	Trierweiler	Rheinland-Palatinate	*Eptesicus serotinus*	1
July 2021	Laubach	Hesse	*Pipistrellus pipistrellus*	6
	Frankfurt	Hesse	*P. pipistrellus*	11
	Hohendrubau	Saxony	*P. pipistrellus*	32
	Biesenthal	Brandenburg	*P. pygmaeus*	1
			*P. pipistrellus*	6
	Marburg	Hesse	*P. pipistrellus*	2
			*P. pipistrellus*	3
			*P. pipistrellus*	9
August 2021	Niederzier	North Rhein-Westphalia	*P. nathusii*	72
	Hatzfeld	Hesse	*P. pipistrellus*	28
			*P. pipistrellus*	1
			*P. pipistrellus*	3
	Niederzier	North Rhein-Westphalia	*P. pipistrellus*	37
	Walkenried	Lower Saxony	*P. pipistrellus*	4
			*P. pipistrellus*	5
Total				224

**Table 2:** *Carios vespertilionis* collected from sick bats in central Germany

Federal state	Locations	North	East	Date	Bat species	No. larvae
Hesse	Mühltal	49.813.743	8.692.766	January 2002	*Pipistrellus pipistrellus*	5
	Messel	49.930.694	8.769.209		*P. pipistrellus*	13
	Fulda	50.557.799	9.656.396	August 2002	*P. pipistrellus*	4
					*P. pipistrellus*	1
					*P. pipistrellus*	2
	Darmstadt	49.858.500	8.661.652	December 2002	*Eptesicus nilssonii*	1
	Korbach	51.246.970	8.804.449	April 2003	*E. nilssonii*	1
	Hilders	50.564.389	10.034.891	August 2003	*Myotis myotis*	3
	Ebersburg	50.429.934	9.806.138	July 2002	*P. pipistrellus*	3
	Hofgeismar	51.482.623	9.390.037	July 2004	*Vespertilio murinus*	3
	Fulda	50.557.799	9.656.396	August 2004	*P. pipistrellus*	7
					*P. pipistrellus*	16
				September 2004	*P. pipistrellus*	2
	Kassel	51.297.149	9.434.745	August 2004	*P. pipistrellus*	1*
		50.557.799	9.656.396		*P. pipistrellus*	7
	Oberaula	50.849.419	9.454.077	October 2004	*P. pipistrellus*	1
	Fulda	50.557.799	9.656.396	February 2006	*P. pipistrellus*	1
				February 2008	*P. pipistrellus*	3
	Eichenzell	50.448.183	9.729.964	March 2009	*P. pipistrellus*	1
Lower Saxony	Hanover	52.365.920	9.694.158	June 2022	*P. pipistrellus*	3
					*M. daubentonii*	4
					*P. pipistrellus*	1
					*P. pipistrellus*	5
					*P. pipistrellus*	38
				July 2022	*P. pipistrellus*	4
				August 2022	*V. murinus*	97
				September 2022	*P. pipistrellus*	2
				December 2022	*P. pipistrellus*	8
				January 2023	*P. pipistrellus*	29
				February 2023	*P. pipistrellus*	29
Total						295

*This tick was in fact a nymph

## Discussion

In the Palearctic Region, bats are generally infested with a low number of ticks (Rupp et al. 2004; Hornok et al. 2017). In the UK, only 26 bats from the 7,606 submitted bats were infested with *C. vespertilionis* larvae (Lv et al. 2018). The distribution of *C. vespertilionis* in central Germany is known (Hutter et al. 2005) and results show that most of the examined bats did not carry any ticks or were only infested with a few ticks (n = 1–5 *C*. *vespertilionis/*bat). However, a few bats carried > 10 ticks. Nine *P*. *pipistrellus* were infested with 11, 13, 16, 28, 29 (2x), 32, 37, and 38 *C*. *vespertilionis* larvae, respectively. One *P*. *nathusii* bat was infested with 72 *C*. *vespertilionis* larvae. The highest number of larvae (n = 97) was collected from one *V*. *murinus*.

A study carried out in Bavaria (southern Germany) showed that generally, 1–2% of the bats is infested with ticks and the number of *C*. *vespertilionis* larvae found on 11 bat species averaged between 2 and 10 ticks (Rupp et al. 2004). There are few reports about bats infested with an uncommonly high number of ticks. Among 51 *P. pipistrellus* examined in Thuringia (Germany) in 2001/2002, three carried 12, 13, and 27 larvae of *C*. *vespertilionis*, respectively (Heddergott 2004). In total 231 *C*. *vespertilionis* larvae were collected from a young *E. nilssonii* in Lower Saxony (Germany), which was severely debilitated and died not long after. This was the highest number of tick larvae found on a bat in Germany (Walter and Rackow 2007).

It is unclear if sick bats are more likely to be infested by ticks or if high tick infestation causes the bats to become unwell. In the present study, the bat infested with 32 ticks appeared very weak, whereas the bat infested with 72 ticks appeared healthy. Ectoparasites such as ticks can cause short- and long-term health issues. Ticks may directly affect bats by sucking their blood, especially when highly infested. The direct impact associated with *C*. *vespertilionis* infestation are minor wounds in the bat’s skin evoked by tick bites that may cause physiological stress and inflammatory responses (del Cacho et al. 1994). Besides that, viruses and bacteria transmitted during the blood meal may have harmful effects on the bats’ health. Studies investigating *C. vespertilionis* (larvae, nymphs, and adults) collected from a wooden bat box harbouring *P. pygmaeus* in Sweden found 24% (22 of 92) of the *C. vespertilionis* specimens to be *Borrelia*-positive, with *Borrelia* sp. CPB1 (Jaenson and Wilhelmsson 2021), a spirochete found before in the UK (Evans et al. 2009) and in France (Socolovschi et al. 2012). Tompa et al. (2023) identified *Rickettsia* species, genetically closely related to *Rickettsia parkeri*, *Rickettsia conorii*, *Rickettsia slovaca*, *Rickettsia sibirica* subsp. *mongolotimonae*, *Rickettsia rickettsii*, and an uncultured *Rickettsia* sp. Several of these species are considered pathogenic to humans, nevertheless most of them are not usually circulating in European tick species. The Sweden studies from Jaenson and Wilhelmsson (2021) and Tompa et al. (2023) (using the same set of samples) concluded that further investigations are needed to confirm *C. vespertilionis’* vector and/or reservoir capacity for the various pathogens. The aim of the current study was to gather more information about the distribution of *C. vespertilionis* on bats in Germany. Therefore, no studies on pathogens were planned and conducted. In the light of the Swedish results, future studies also have to focus on bacterial pathogens in bat soft tick to elucidate their potential to support the circulation of zoonotic pathogens.

During the present study, only larvae of *C. vespertilionis* were collected, except one nymph. *Carios vespertilionis* is frequently found in caves and its larvae are found on bats, with few exceptions. This can be explained by the long feeding time of larvae from a few days up to 14 or even 31 days. Nymphs and adult ticks, on the other hand, feed very quickly for only a short period of < 1 h (Walter and Kock 1985; Hoogstraal 1956; Walter 1992).

In a recent review (Sándor et al. 2021), 44 bat species were listed as hosts of soft ticks. Among them, *C*. *vespertilionis* had the most diverse host spectrum with 42 bat species in total. In the present study, *C*. *vespertilionis* larvae were found on a wide range of bat species, mainly on *P*. *pipistrellus* (common pipistrelle). These results support findings from previous studies where this bat species was identified as the main host in Germany. However, *C*. *vespertilionis* was also found on eight other bat species: *N*. *leisleri* (Leisler’s bat), *E*. *nilssonii* (northern bat), *E*. *serotinus* (serotine bat), *M*. *daubentonii* (Daubenton’s bat), *M*. *myotis* (greater mouse-eared bat), *P*. *nathusii* (Nathusius’s pipistrellus), *P*. *pygmaeus* (soprano pipistrelle) and *V*. *murinus* (parti-coloured bat). In Lv et al. (2018), of the nine bat species included in the study three species – *P. pipistrellus*, *Plecotus auritus*, and *M. daubentoniid* – carried *C. vespertilionis* larvae, with *P. pipistrellus* being the most infested. In Sweden, *C*. *vespertilionis* have been recorded from two bat species – *E. nilssoni* and *P. pipistrellus* – and from dog (*Canis familiaris*) (Jaenson et al. 1994), whereas in Norway, four bat species – *M. mystacinus*, *M. daubentoni*, *P. pipistrellus*, and *E. nilssoni* – are known as hosts of this tick (Mehl 1983).

In the latest overview about the distribution of *C*. *vespertilionis* in Germany (Rubel et al. 2023), no georeferenced data points were reported in Saxony and Saxony-Anhalt federal states, but one in Thuringia and several in Brandenburg and Mecklenburg-West Pomerania. Here, the presence of *C*. *vespertilionis* in eastern Saxony was documented, which fills the gap between central German Federal States and adjacent Poland, where this tick species has been reported (Siuda et al. 2009; Mierzyński et al. 2018).

The currently known distribution of *C*. *vespertilionis* in Germany has been confirmed for the following federal states: Lower Saxony (Scheffler and Hiller 2010), Brandenburg (Cornely and Schultz 1992), North Rhine-Westphalia, Hesse, and Rhineland-Palatinate (Walter 1992). When reporting *C*. *vespertilionis* for a specific location, the collection time should be considered, as some bats have a wide distribution area. *Pipistrellus nathusii*, for example, migrates long distances of up to 2,486 km in one direction (Vasenkov et al. 2022). Migrating bat species (e.g., *N. leisleri*, *N. noctula*, *P. nathusii*, *V. murinus*) can travel > 4000 km in total each year during their flights between their summer and winter habitats (Hutterer et al. 2005).

Regarding the winter activity of *C*. *vespertilionis*, there are only two reports on ticks found on bats. One was in January 1985 and the other in February 1987, both in Baden-Wuerttemberg, southern Germany (Walter 1992). On top of those reports a few specimens of *C*. *vespertilionis* were found in bat faeces. In the present study *C*. *vespertilionis* larvae were collected during the winter months (December, January, and February) in the federal states Hesse and Lower Saxony. Larvae, nymphs and adults were collected in summer (June, July, and August 2015 and 2018) using a water-filled tray placed bellow a wood bat box harbouring an estimated number of 250–500 adult females and young *P. pygmaeus* (Jaenson and Wilhelmsson 2021). In the UK, larvae and nymphs where collected from *P. pipistrellus* in May and July and in material from roost in house in July (Arthur 1963).

## Conclusions

There are still considerable knowledge gaps regarding the presence of several tick species in Germany, especially concerning rare species, such as *C. vespertilionis*. This study supplies more detailed information for some federal states in central Germany, but additional studies are warranted on *C. vespertilionis* presence, density, and evaluation of public and veterinary risks.
